# Transactions of the New York State Medical Association for the Year 1887

**Published:** 1888-07

**Authors:** 


**Transactions of the New York State Medical Association for the Year 1887.** Edited by Alfred L. Carroll, M. D. J. H. Vail & Co. New York: 1888.

Never have the transactions of this Association, and we add, of any other New York State society, been published in a more attractive form. The lithographs and colored plates are excellent, while the published papers, without exception, show careful thought and often much original research. This book should find a place in every physician's library.

